# Polymorphisms in the circadian expressed genes *PER3* and *ARNTL2* are associated with diurnal preference and *GNβ3* with sleep measures

**DOI:** 10.1111/jsr.12144

**Published:** 2014-03-17

**Authors:** Michael J Parsons, Kathryn J Lester, Nicola L Barclay, Simon N Archer, Patrick M Nolan, Thalia C Eley, Alice M Gregory

**Affiliations:** 1 MRC Harwell, Harwell Science and Innovation CampusOxfordshire, UK; 2 Institute of Psychiatry, King's College LondonLondon, UK; 3 Department of Psychology, Northumbria Centre for Sleep Research, Northumbria UniversityNewcastle upon Tyne, UK; 4 Surrey Sleep Research Centre, University of SurreyGuilford, UK; 5 Department of Psychology, Goldsmiths, University of LondonLondon, UK

**Keywords:** circadian expressed genes, genetic association, single nucleotide polymorphisms, sleep duration, sleep quality

## Abstract

Sleep and circadian rhythms are intrinsically linked, with several sleep traits, including sleep timing and duration, influenced by both sleep homeostasis and the circadian phase. Genetic variation in several circadian genes has been associated with diurnal preference (preference in timing of sleep), although there has been limited research on whether they are associated with other sleep measurements. We investigated whether these genetic variations were associated with diurnal preference (Morningness–Eveningness Questionnaire) and various sleep measures, including: the global Pittsburgh Sleep Quality index score; sleep duration; and sleep latency and sleep quality. We genotyped 10 polymorphisms in genes with circadian expression in participants from the G1219 sample (*n* = 966), a British longitudinal population sample of young adults. We conducted linear regressions using dominant, additive and recessive models of inheritance to test for associations between these polymorphisms and the sleep measures. We found a significant association between diurnal preference and a polymorphism in period homologue 3 (*PER3*) (*P* < 0.005, recessive model) and a novel nominally significant association between diurnal preference and a polymorphism in aryl hydrocarbon receptor nuclear translocator-like 2 (*ARNTL2*) *(P* < 0.05, additive model). We found that a polymorphism in guanine nucleotide binding protein beta 3 (*GNβ3*) was associated significantly with global sleep quality (*P* < 0.005, recessive model), and that a rare polymorphism in period homologue 2 (*PER2*) was associated significantly with both sleep duration and quality (*P* < 0.0005, recessive model). These findings suggest that genes with circadian expression may play a role in regulating both the circadian clock and sleep homeostasis, and highlight the importance of further studies aimed at dissecting the specific roles that circadian genes play in these two interrelated but unique behaviours.

## Introduction

Circadian rhythms are daily cycles controlled by a central hypothalamic pacemaker and affect a wide range of physiological and behavioural, outputs including the sleep–wake cycle (Franken and Dijk, 2009). This central pacemaker is known to be governed by a complex molecular clock, which consists of various transcriptional feedback loops and output systems (reviewed in Zhang and Kay, 2010). Previous research has determined a number of the genes involved in these transcriptional feedback loops that drive the central pacemaker (the clock genes) and numerous other genes whose expression are rhythmically regulated by these core clock genes (reviewed in Zhang and Kay, 2010).

Diurnal preference, an individual's preference of timing of the sleep–wake cycle, is influenced by both the circadian clock and by sleep homeostatis (Kerkhof, 1991; Mongrain *et al*., 2004, 2006; Schmidt *et al*., 2009; Taillard *et al*., 2003). Diurnal preference and certain sleep measures, including sleep quality and duration, vary across individuals. Twin studies suggest that genetic influences partially underlie the variation seen for diurnal preference, sleep quality and sleep duration (Barclay *et al*., 2010a,b; Partinen *et al*., 1983). Previously, genetic variation in several clock genes [circadian locomotor output cycles kaput (*CLOCK*), period homologue (*PER*)*1*, *PER2*, *PER3*] has been shown to be associated with diurnal preference in the general population (Archer *et al*., 2010; Carpen *et al*., 2006; Katzenberg *et al*., 1998; Lee *et al*., 2007, 2011). Genetic variation in guanine nucleotide binding protein beta 3 (*GNβ3*), which has circadian expression in the mouse pituitary (Hughes *et al*., 2009), has also been associated with diurnal preference (Johansson *et al*., 2004). Similarly, other studies have suggested that variation in other circadian genes, aryl hydrocarbon receptor nuclear translocator-like 2 (*ARNTL2*) and D site of albumin promoter (albumin D-box) binding protein (*DBP*), may affect specific circadian outputs, such as daily changes in mood (Shi *et al*., 2008).

Sleep and circadian rhythms are intrinsically linked, with a number of sleep traits, including sleep timing and duration, influenced by both sleep homeostasis and the circadian phase (Borbely, 1982). Given the influences of the circadian clock on sleep behaviours, we hypothesized that the same single nucleotide polymorphisms (SNPs) that had been associated with diurnal preference could also be associated with additional subjective sleep measures. We thus attempted to both replicate the associations of these SNPs in genes with circadian expressionwith diurnal preference and to further investigate whether they were associated with specific subjective sleep measures in the G1219 sample. We limited our investigations to those SNPs that were associated previously with diurnal preference and circadian outputs, e.g. worse evening mood (summarized in Table 1), including a rare, unpublished F-box and leucine-rich repeat protein 3(*FBXL3*) SNP that was found previously to be associated with extreme values of diurnal preference (S. N. Archer, unpublished data).

**Table 1:** Summary of Investigated single nucleotide polymorphisms (SNPs)

Gene	SNP ID	Allele	Genotypic frequency			Gene region	Related phenotype	Citation

			M/M	M/m	m/m			
ARNTL2	rs922270	T/C	690 (72.9)	240 (25.3)	17 (1.8)	Intronic region	Worse evening mood	Shi *et al*. (2008)
CLOCK	rs2070062	T/C	530 (56.1)	341 (36.1)	73 (7.7)	3′ UTR region	Diurnal preference	Pedrazzoli *et al*. (2007)
DBP	rs3848543	C/T	673 (73.3)	231 (25.2)	14 (1.5)	Intronic region	Worse evening mood	Shi *et al*. (2008)
FBXL3	825679097	G/T	939 (99.8)	2 (0.2)	0 (0)	Coding (Gly/Val)	Diurnal preference	S. N. Archer (unpublished data)
GNβ3	rs5443	C/T	466 (49.4)	387 (41)	91 (9.6)	Affects splicing	Diurnal preference	Johansson *et al*. (2004)
PER1	rs2735611	T/C	688 (73.7)	224 (24)	22 (2.4)	Coding (silent)	Diurnal preference	Carpen *et al*. (2006)
PER2	rs934945	G/A	604 (64.2)	295 (31.4)	42 (4.5)	Coding (Gly/Glu)	Diurnal preference	Lee *et al*. (2007) and Carpen *et al*. (2006)
PER2	rs2304672	C/G	796 (84.4)	144 (15.3)	3 (0.3)	Promoter region	Diurnal preference	Carpen *et al*. (2006) and Lee *et al*. (2011)
PER3	rs2797687	G/T	611 (67.1)	266 (29.2)	33 (3.6)	Promoter region	DSPD	Archer *et al*. (2010)
PER3	rs10462020	G/T	606 (64.3)	274 (29.1)	63 (6.7)	Coding (Val/Gly)	Diurnal preference and DSPD	Johansson *et al*. (2003); Ebisawa *et al*. (2001)

ARNTL2, aryl hydrocarbon receptor nuclear translocator-like 2; CLOCK, circadian locomotor output cycles kaput; DBP, D site of albumin promoter (albumin D-box) binding protein; PER, period homologue (PER1, PER2, PER3); FBXL3, F-box and leucine-rich repeat protein 3; GNβ3, guanine nucleotide binding protein beta 3; DSPD, delayed sleep phase disorder.

The genotypic frequencies for the 10 SNPs that varied in the G1219 sample are listed in this table. The allele lists the major allele first (M) and minor allele second (m). The genotypic frequencies relate to the major and minor alleles. Additional information is listed for each SNP, including its position relative to the gene and the related phenotype.

## Materials and Methods

### Participants

The participants in this study come from the G1219 and G1219 Twins longitudinal studies (McAdams *et al*., 2013). The G1219 sample comprises the adolescent offspring of adults from a large-scale population-based study (Eley *et al*., 2004), while the G1219 Twins sample comprises randomly selected twin sets born between 1985 and 1988 (McAdams *et al*., 2013). Informed consent was obtained from parents/guardians of all adolescents aged <16 years, and from the adolescents themselves when ≥16 years old. Ethical approval for different stages of this study has been provided by the Research Ethics Committees of the Institute of Psychiatry, South London and Maudsley NHS Trust, and Goldsmiths, University of London.

This study focuses exclusively on wave 4, comprising 1556 individuals (collected in 2007) as, at the time of analyses, subjective sleep data were available at this wave only. Individuals were selected for inclusion if sleep measures were available and DNA had been collected. The total sample was 1130 (73% of those participating at wave 4). For monozygotic (MZ) twin pairs, data from only one individual, selected at random, was included in the analyses. The analyses are based on sleep data and DNA from 952 wave four participants [mean age = 20.3 years, standard deviation (SD) = 1.77, age range 18–27 years; 61.0% female] consisting of 562 dizygotic (DZ) individuals, 180 MZ individuals and 210 individuals from sibling pairs.

### Measures

#### Diurnal preference

The Morningness–Eveningness Questionnaire (MEQ, Horne and Oostburg, 1976) measured diurnal preference. The MEQ is a 19-item self-report questionnaire which assesses individual preference for the timing of a number of diurnal activities, sleeping habits, hours of peak performance and times of ‘feeling best’ and maximum alertness. Individual items are rated on a 4- or 5-point scale and the total score ranges from 16 to 86. Higher scores indicate a greater ‘morningness’ preference. For further details of the validity of the MEQ in the G1219 sample, see elsewhere (Barclay *et al*., 2010a). The MEQ score had a mean of 48.6 (SD = 8.2).

#### Sleep quality

Sleep quality was assessed using the Pittsburgh Sleep Quality Index (PSQI; Buysse *et al*., 1989), which is a widely used questionnaire measure containing 18 items assessing sleep over the previous month. Global scores range from 0 to 21, with higher scores indicating poorer sleep quality. For further details of the validity of the MEQ in the G1219 sample, see elsewhere (Barclay *et al*., 2011). The global PSQI mean score was 5.9 (SD = 3.1).

In addition to the global score, three measures of sleep (sleep duration, sleep latency and sleep quality) were assessed using the PSQI. Sleep duration was measured using question 4 of this questionnaire: ‘During the past month, how many hours of actual sleep did you get at night?’. Sleep duration ranged from 2 to 12 h with a mean of 7.5 h (SD = 1.2). Sleep latency was measured using question 2 of this questionnaire: ‘During the past month, how long (in min) has it usually taken you to fall asleep each night?’. Sleep latency ranged from 0 to 240 min and had a mean of 27.8 (SD = 23.3). Sleep quality was measured using question 6 of this questionnaire: ‘During the past month, how would you rate your sleep quality overall?’, and was scored: very good = 0; fairly good = 1; fairly bad = 2; and very bad = 3. Sleep quality had a mean score of 1.13 (SD = 0.71).

### DNA extraction and genotyping

Cheek swab kits were posted to participants in order to collect DNA, primarily during wave 4. The DNA extractions are described elsewhere (Barclay *et al*., 2011). Ten single nucleotide polymorphisms (SNPs) were genotyped from a number of genes with circadian expression (see Table 1). The FBXL3_1362G/T SNP that was found previously to be associated with extreme values of diurnal preference (S. N. Archer, unpublished data) was submitted to dbSNP and has the SNP submission number: 825679097. All the genotyping assays were performed by KBioscience (http://www.lgcgenomics.com/) using KASPar chemistry (for more details see: http://www.lgcgenomics.com/genotyping/kasp-genotyping-reagents/kasp-overview/). Blind duplicates and Hardy–Weinberg equilibrium (HWE) tests were used as quality control tests. Linkage disequilibrium and HWE were calculated using the Haploview program (Barrett *et al*., 2005). The *PER3* SNP rs10462020 failed to reach HWE in the total sample (*χ*^2^ = 16.1, *P* = 0.0001), but was in HWE in the male subset (*P* < 0.05). All other SNPs were in HWE (*P* < 0.05).

### Statistical analyses

All analyses were performed in stata (StataCorp., 2002). Linear regressions were conducted to model the main effect of the SNPs on diurnal preference, global PSQI sleep quality, sleep duration, sleep latency and sleep quality. Age and sex were first entered into the regression models, followed by main effects of genotype. We investigated the three non-independent models of inheritance: additive, dominant and recessive (Lewis and Knight, 2012). To investigate the combined effects of SNPs on specific sleep measures, we also conducted regression analyses for each sleep phenotype adding all 10 of the SNPs we investigated into the model simultaneously. So that we could determine additionally if the significant individual associations were independent of each other, we varied the model of inheritance included in the combined analysis for each measure investigated. For each measure we included the model of inheritance that was most significant for each SNP from the individual analysis; for those SNPs with no significant model of inheritance for that measure we used the additive model of inheritance. As our sample included related individuals, all analyses were corrected conservatively for the non-independence of the twin/sibling observations using the ‘*robust*’ *cluster* command in stata, as is standard in analyses of this type (see Rogers, 1993). This analysis should correct for any failures to reach the assumptions that are required for using linear models, such as linearity, independence, normality and homoscedasticity. If these assumptions are met, then a standard linear regression model should account for differences in group size across the genotype groups. We applied a Bonferroni correction to control for multiple testing independently for each of the sleep measures. We adjusted the *P*-value by using the total number of SNPs investigated for each trait, thus the corrected *P*-values for diurnal preference, global PSQI sleep quality, sleep duration, sleep latency and sleep quality are: corrected *P* = 0.05/10 = 0.005. We did not apply corrections for multiple testing for the number of inheritance models that we ran, as these tests were not independent of each other, and instead tested related hypotheses. Similarly, we did not apply further multiple testing corrections across all the phenotypes investigated, as a number of sleep measures are at least partially correlated and thus are not fully independent tests. If we were to correct stringently for all sleep measures investigated, then we would require a Bonferroni-adjusted *P*-value of 0.001. None of the analyses reported here would survive this correction, but this would be a very conservative correction given that the sleep measures are not completely independent of each other.

## Results

The genotype counts and percentages for the 10 SNPs investigated are summarized in Table 1. Table 2 summarizes the means and standard deviations for each measure by genotype.

**Table 2:** Means and standard deviations for all sleep measures by genotype

Gene	SNP ID	Allele	Diurnal preference	Global PSQI	Sleep duration	Sleep latency	Sleep quality
ARNTL2	rs922270	TT	48.4 (8.2)	5.9 (3.2)	7.5 (1.2)	28.7 (23.2)	1.2 (0.7)
		TC	48.8 (8.2)	5.6 (2.9)	7.5 (1.1)	26.1 (24.3)	1 (0.7)
		CC	52.6 (6.6)	6 (2.6)	7.6 (0.9)	20.7 (13.3)	1.1 (0.6)
		Total *n*	945	927	932	934	941
CLOCK	rs2070062	TT	48.6 (8.2)	5.8 (3.2)	7.5 (1.2)	28.3 (24.6)	1.1 (0.7)
		TG	48.7 (8.1)	5.9 (2.9)	7.4 (1.2)	26.9 (21.2)	1.1 (0.7)
		GG	47.7 (9.1)	5.8 (3.1)	7.4 (1.2)	25.7 (16.3)	1.2 (0.7)
		Total *n*	942	924	929	931	938
DBP	rs3848543	CC	48.7 (8.3)	5.9 (3.1)	7.4 (1.2)	28.8 (24.9)	1.1 (0.7)
		CT	48.4 (7.8)	5.5 (3.2)	7.6 (1.2)	24.9 (18.2)	1.1 (0.7)
		TT	46.2 (9.4)	6.3 (4)	6.9 (1.3)	28.9 (25.7)	1.2 (0.8)
		Total *n*	916	899	903	905	913
FBXL3	825679097	GG	48.5 (8.2)	5.9 (3.1)	7.5 (1.2)	27.8 (23.5)	1.1 (0.7)
		GT	52 (11.3)	5 (1.4)	8.3 (1.1)	22.5 (10.6)	1 (0.0)
		Total *n*	938	920	925	927	934
GNβ3	rs5443	CC	48.3 (8.2)	5.9 (3)	7.5 (1.2)	27.3 (20.3)	1.2 (0.7)
		CT	48.6 (8.5)	6 (3.3)	7.4 (1.3)	28.6 (27)	1.1 (0.8)
		TT	49.5 (7.4)	5.1 (2.8)	7.7 (1)	26.8 (21.5)	1 (0.7)
		Total *n*	942	924	929	931	938
PER1	rs2735611	TT	48.7 (8.3)	5.9 (3.1)	7.5 (1.2)	28 (23.9)	1.1 (0.7)
		TC	48.2 (8)	5.8 (3)	7.6 (1.2)	27.2 (21.9)	1.2 (0.7)
		CC	48.8 (6.4)	6.3 (3.8)	7 (1.1)	31.7 (28.2)	1 (0.7)
		Total *n*	932	914	919	921	928
PER2	rs934945	GG	48.6 (8.4)	5.8 (3.1)	7.5 (1.2)	27.6 (24.1)	1.1 (0.7)
		GA	48.6 (7.9)	6.1 (3.1)	7.4 (1.2)	29.1 (22.9)	1.2 (0.7)
		AA	49.8 (9.2)	6.1 (3.4)	7.3 (1.1)	24.9 (15.9)	1.2 (0.8)
		Total *n*	939	922	927	928	935
PER2	rs2304672	CC	48.7 (8.1)	5.8 (3.1)	7.5 (1.2)	27 (21.2)	1.1 (0.7)
		CG	48 (8.7)	6 (3.3)	7.5 (1.4)	31.5 (30.2)	1.1 (0.7)
		GG	42.3 (7.5)	4.3 (1.5)	8.2 (0.3)	21.7 (7.6)	1 (0)
		Total *n*	941	923	928	930	937
PER3	rs2797687	CC	48.4 (8.5)	5.9 (3.1)	7.5 (1.2)	28.1 (23.2)	1.1 (0.7)
		CA	48.6 (7.6)	5.8 (3.1)	7.5 (1.3)	27.4 (22.6)	1.2 (0.7)
		AA	49.4 (8.4)	5.8 (2.8)	7.3 (1)	26.1 (23.8)	1.1 (0.6)
		Total *n*	908	891	895	898	905
PER3	rs10462020	TT	48.2 (8.3)	5.9 (3.1)	7.4 (1.2)	28.1 (22.9)	1.1 (0.7)
		TG	48.5 (8)	6 (3.2)	7.6 (1.3)	27.2 (21.1)	1.2 (0.7)
		GG	51.3 (7.7)	5.3 (2.7)	7.6 (1.2)	29.7 (34.9)	1 (0.6)
		Total *n*	941	923	928	930	937

ARNTL2, aryl hydrocarbon receptor nuclear translocator-like 2; CLOCK, circadian locomotor output cycles kaput; DBP, D site of albumin promoter (albumin D-box) binding protein; PSQI, Pittsburgh Sleep Quality Index; PER, period homologue (PER1, PER2, PER3); FBXL3, F-box and leucine-rich repeat protein 3; GNβ3, guanine nucleotide binding protein beta 3.

The mean scores with standard deviations (in parentheses) are listed for all sleep measures by genotype, including: diurnal preference (range of 16–86, a higher score indicates greater preference for morningness), global PSQI score (range from 0 to 21, with higher scores indicating poorer overall sleep quality); sleep duration (h), sleep latency (min) and sleep quality (range 0–3, higher scores indicate poorer sleep quality).

### Diurnal preference

**Figure 1:**
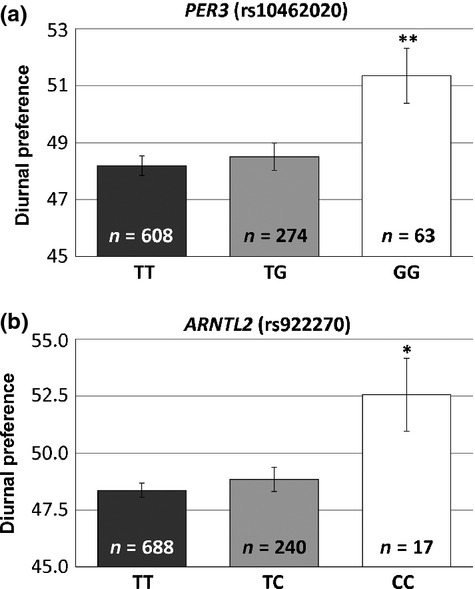
Period homologue 3 (PER3) and aryl hydrocarbon receptor nuclear translocator-like 2 (ARNTL2) single nucleotide polymorphisms (SNPs) are associated with diurnal preference.

There was a significant association between the *PER3* SNP rs10462020 genotype and the mean diurnal preference score using a recessive model of inheritance (*P* = 0.003) (see Table 3 for a summary of the linear regression scores). These results indicate that the GG individuals (*n* = 63) had a higher diurnal preference score, and thus an increased morning preference (mean = 51.3, SD = 7.7), than the TT and TG individuals (mean = 48.3, SD = 8.2) (see Fig. 1a). There was also a nominally significant association between this SNP and mean diurnal preference using an additive model, *P* = 0.02). As this SNP was not in HWE in the total sample, but was in HWE in the male subsample, we repeated the regression analyses in the male subsample. We found that there was still a significant association between rs10462020 genotype and the mean diurnal preference in the male subsample using a recessive model of inheritance (β = 5.2, *P* = 0.003).

**Table 3:** Standardized regression coefficients β (SE) from linear regression analyses for main effects of genotype on all sleep measures

Gene	SNP		Diurnal reference	Global PSQI	Sleep duration	Sleep latency	Sleep quality
ARNTL2	rs922270	Additive	0.76 (0.53)	−0.23 (0.2)	0.02 (0.08)	−2.96 (1.47)*	−0.1 (0.05)*
		Recessive	3.72 (1.55)*	0.07 (0.56)	0.06 (0.21)	−7.19 (3.11)*	−0.02 (0.14)
		Dominant	0.59 (0.59)	−0.28 (0.23)	0.02 (0.09)	−2.97 (1.76)	−0.12 (0.05)*
CLOCK	rs2070062	Additive	−0.27 (0.44)	0.02 (0.17)	−0.08 (0.06)	−1.37 (1.09)	0.03 (0.04)
		Recessive	−0.74 (1.11)	0.05 (0.4)	−0.1 (0.16)	−2.42 (2.16)	0.06 (0.09)
		Dominant	−0.23 (0.53)	0.02 (0.21)	−0.1 (0.08)	−1.55 (1.49)	0.04 (0.05)
DBP	rs3848543	Additive	−0.54 (0.56)	−0.31 (0.23)	0.03 (0.09)	−2.99 (1.51)*	−0.06 (0.05)
		Recessive	−2.58 (2.33)	0.44 (0.94)	−0.63 (0.33)	1.02 (6.65)	0.09 (0.2)
		Dominant	−0.45 (0.6)	−0.41 (0.25)	0.09 (0.09)	−3.65 (1.59)*	−0.08 (0.06)
FBXL3	825679097	Additive	2.33 (5.88)	−1.1 (0.82)	0.81 (0.44)	−4.76 (5.03)	−0.15 (0.04)**
		Dominant	2.33 (5.88)	−1.1 (0.82)	0.81 (0.44)	−4.76 (5.03)	−0.15 (0.04)**
GNβ3	rs5443	Additive	0.53 (0.4)	−0.23 (0.15)	0.04 (0.06)	0.35 (1.11)	−0.07 (0.03)*
		Recessive	0.95 (0.81)	−0.9 (0.32)**	0.24 (0.11)*	−1.03 (2.34)	−0.2 (0.08)*
		Dominant	0.59 (0.56)	−0.09 (0.21)	0 (0.08)	0.96 (1.6)	−0.06 (0.05)
PER1	rs2735611	Additive	−0.28 (0.49)	0 (0.22)	0.01 (0.08)	−0.08 (1.58)	0.02 (0.05)
		Recessive	−0.13 (1.38)	0.38 (0.88)	−0.49 (0.27)	3.96 (6.2)	−0.14 (0.17)
		Dominant	−0.34 (0.58)	−0.04 (0.23)	0.06 (0.09)	−0.51 (1.69)	0.03 (0.05)
PER2	rs934945	Additive	0.29 (0.47)	0.28 (0.19)	−0.13 (0.07)	0.24 (1.24)	0.04 (0.04)
		Recessive	1.43 (1.34)	0.31 (0.57)	−0.21 (0.17)	−3.32 (2.64)	0.09 (0.13)
		Dominant	0.15 (0.56)	0.34 (0.22)	−0.14 (0.08)	0.96 (1.61)	0.05 (0.05)
PER2	rs2304672	Additive	−1.16 (0.75)	0.04 (0.28)	0.01 (0.11)	3.93 (2.44)	−0.04 (0.06)
		Recessive	−5.66 (3.68)	−1.47 (0.79)	0.78 (0.15)**	−6.04 (4)	−0.12 (0.03)**
		Dominant	−1.1 (0.78)	0.08 (0.3)	−0.01 (0.12)	4.34 (2.58)	−0.04 (0.06)
PER3	rs2797687	Additive	0.32 (0.48)	−0.06 (0.18)	−0.01 (0.07)	−0.85 (1.37)	0.01 (0.04)
		Recessive	0.81 (1.38)	−0.09 (0.49)	−0.25 (0.17)	−1.95 (4.23)	−0.04 (0.11)
		Dominant	0.32 (0.56)	−0.06 (0.22)	0.02 (0.09)	−0.87 (1.59)	0.02 (0.05)
PER3	rs10462020	Additive	1.02 (0.43)*	−0.09 (0.16)	0.11 (0.06)	0.02 (1.52)	−0.01 (0.04)
		Recessive	2.99 (1.01)**	−0.6 (0.35)	0.14 (0.15)	1.85 (4.41)	−0.11 (0.08)
		Dominant	0.87 (0.55)	0.01 (0.21)	0.14 (0.08)	−0.47 (1.63)	0.02 (0.05)

ARNTL2, aryl hydrocarbon receptor nuclear translocator-like 2; CLOCK, circadian locomotor output cycles kaput; DBP, D site of albumin promoter (albumin D-box) binding protein; PSQI, Pittsburgh Sleep Quality Index; PER, period homologue (PER1, PER2, PER3); FBXL3, F-box and leucine-rich repeat protein 3; GNβ3, guanine nucleotide binding protein beta 3; SE, standard error.

The table presents the standardized regression coefficients for linear regression analyses using additive, recessive and dominant models of inheritance for the various sleep measures. Significant results are highlighted in bold type.

* Nominally significant results (*P* < 0.05).

** Significant result following multiple testing correction (*P* < 0.005).

There was also a nominally significant association between the *ARNTL2* SNP rs922270 and mean diurnal preference score using a recessive model (*P* = 0.02) (see Fig. 1b). These results indicate that the CC individuals (*n* = 17) had a higher diurnal preference, and thus an increased morning preference (mean = 52.6, SD = 6.6), than the TT and TC individuals (mean = 48.5, SD = 8.2).

In the combined analysis (including all 10 SNPs investigated), the *PER3* SNP remained significant (β = 3.3, *P* = 0.003, recessive model), although the *ARNTL2* SNP did not (β = 2.8, *P* = 0.013, recessive model). We also found two novel associations not seen in the individual analysis: the rare *FBXL3* SNP 825679097 (β = 11.7, *P* < 0.0001, additive model) and the *GNβ3* SNP rs5443 (β = 0.9, *P* = 0.04, additive model).

### Global PSQI score

**Figure 2:**
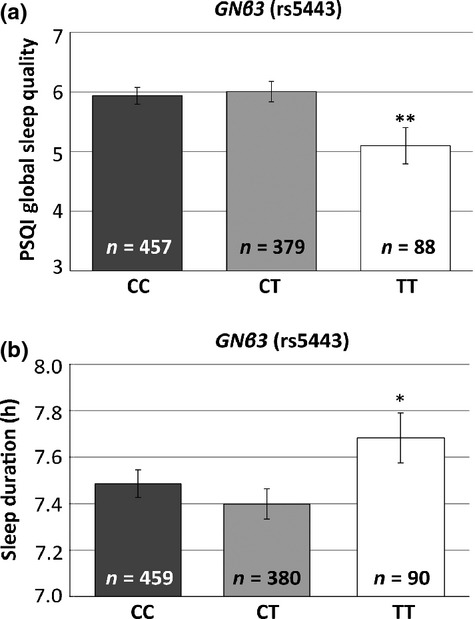
Guanine nucleotide binding protein beta 3 (*GNβ3*) is associated with variations in global sleep quality and sleep duration in recessive models.

There was a significant association between the *GNβ3* SNP rs5443 and the global PSQI score using a recessive model (*P* = 0.005) (see Fig. 2a). These results indicate that the TT individuals (*n* = 88) had a lower global PSQI score, and thus less overall sleep disturbance (mean = 5.10, SD = 2.8), than the CC and CT individuals (mean = 5.97, SD = 3.1). In the combined analysis (including all 10 SNPs investigated), this SNP was still nominally significant in a recessive model (β = −0.7, *P* = 0.04).

### Sleep duration

There was a significant association between the *PER2* SNP rs2304672 and sleep duration using a recessive model (*P <* 0.001). These results indicate that the rare GG individuals (*n* = 3) had a longer sleep duration (mean = 8.2 h, SD = 0.3) than the CC and CG individuals (mean = 7.5 h, SD = 1.2).

There was also a nominally significant association between the *GNβ3* SNP rs5443 and sleep duration using a recessive model (*P =* 0.03) (see Fig. 2b). These results indicate that the TT individuals (*n* = 88) had a longer sleep duration (mean = 7.7 h, SD = 1.0) than the CC and CT individuals (mean = 7.4 h, SD = 1.2).

In the combined analysis (including all 10 SNPs investigated), the associations with both the *PER2* SNP rs2304672 (β = 0.6, *P* < 0.001, recessive) and the *GNβ3* SNP rs5443 (β = 0.29, *P* = 0.03, recessive) remained mainly unchanged. We additionally found a novel association with the rare *FBXL3* SNP 825679097 (β = 1.3, *P* < 0.0001, additive model).

### Sleep latency

**Figure 3:**
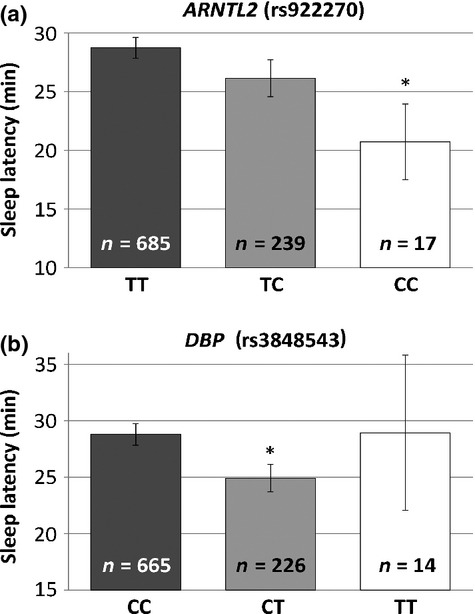
Aryl hydrocarbon receptor nuclear translocator-like 2 (ARNTL2) and D site of albumin promoter (albumin D-box) binding protein (DBP) are associated with variations in sleep latency.

There was a nominally significant association between the *ARNTL2* SNP rs922270 and sleep latency in both an additive model (*P =* 0.045) and a recessive model (*P =* 0.02) (see Fig. 3a). These results indicate that the CC individuals (*n* = 17) had a lower sleep latency, thus taking a shorter period of time to fall asleep (mean = 20.7 min, SD = 13.3) than the TC (mean = 26.1 min, SD = 24.3) and TT individuals (mean = 28.7 min, SD = 23.2).

There was also a nominally significant association between the *DBP* SNP rs3848543 and sleep latency in both an additive model (*P =* 0.047) and a dominant model (*P =* 0.02) (see Fig. 3b). These results indicate that the CT individuals (*n* = 231) had a lower sleep latency, thus taking a shorter period of time to fall asleep (mean = 24.9 min, SD = 18.2) than the CC (mean = 28.8 min, SD = 24.9) and TT individuals (mean = 28.9 min, SD = 25.7). In the combined analysis (including all 10 SNPs investigated), we found only a nominal trend towards significance for the *DBP* SNP (β = −2.7, *P* = 0.09, additive model).

### Sleep quality

There was a significant association between the *FBXL3* SNP 825679097 and sleep quality using an additive model (*P <* 0.001). These results indicate that the rare GT individuals (*n* = 2) had a lower sleep quality score, thus better sleep quality (mean = 1.0, SD = 0.0), than the GG individuals (mean = 1.1, SD = 0.7).

There was also a significant association between the *PER2* SNP rs2304672 and sleep quality using a recessive model (*P <* 0.001). These results indicate that the rare GG individuals (*n* = 3) had a lower sleep quality score, thus better sleep quality (mean = 1.0, SD = 0.0), than the CC and CG individuals (mean = 1.1, SD = 0.7).

**Figure 4:**
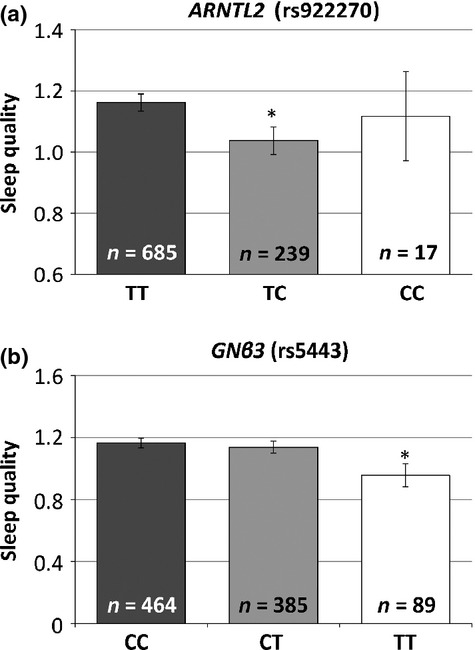
Aryl hydrocarbon receptor nuclear translocator-like 2 (ARNTL2) and guanine nucleotide binding protein beta 3 (*GNβ3*) are associated with variations in sleep quality.

There were nominally significant associations between the *ARNTL2* SNP rs922270 and sleep quality in both additive (*P* = 0.03) and dominant models (*P =* 0.02) and the *GNβ3* SNP rs5443 and sleep quality in both additive (*P =* 0.03) and recessive models (*P =* 0.01) (see Fig. 4a,b). The *ARNTL2* CC individuals (*n* = 17) and the *GNβ3* TT individuals (*n* = 89) had lower sleep quality scores, and thus better sleep quality (mean = 1.1, SD = 0.6 and mean = 1.0, SD = 0.7), respectively, than the other genotypes (see Table 2 for details).

In the combined analysis (including all 10 SNPs investigated), only the rare *FBXL3* SNP 825679097 was nominally significant (β = −0.14, *P* = 0.04, additive model).

## Discussion

### Diurnal preference

We found a significant association of a *PER3* SNP rs10462020 with diurnal preference. This finding is in line with a wide range of studies showing that genetic variation in the *PER3* gene is associated with diurnal preference (Archer *et al*., 2003; Ebisawa *et al*., 2001), including a study that also found the G allele to be associated with increased morning preference (Johansson *et al*., 2003). This SNP is also part of a haplotype associated with delayed sleep phase syndrome (Ebisawa *et al*., 2001). Taken together, these studies suggest that this gene may play a role in regulating diurnal preference.

The *PER3* SNP was found to be significantly out of HWE in the total sample, but not in the male subsample. As the association between this SNP and diurnal preference was significant in both the total sample and the male subsample, this suggests that this association is unlikely to be due to genotyping error. Failure to reach HWE could be due to a number of other factors, including non-random mating, which occurs with morningness–eveningness (Randler and Kretz, 2011).

The mechanism by which the *PER3* gene modulates diurnal preference is uncertain, as diurnal preference can be affected by both circadian rhythms and sleep homeostasis. Period genes act as negative regulators of the molecular clock, with *Per1* and *Per2* playing more central roles than *Per3*. *Per1* and *Per2* knockout mice have a shorter circadian period, while the *Per3* knockout mice have only subtle (Bae *et al*., 2001) or no differences in circadian period (Van der Veen and Archer, 2010). Additionally, while *Per1/Per2* double knockout mice are arrhythmic in constant darkness, *Per1/Per3* or *Per2/Per3* double knockout mice are not (Bae *et al*., 2001). More recently, the *PER3* gene has also been shown to play a role in sleep homeostasis in humans (Viola *et al*., 2007) and its orthologue plays the same role in mice (Hasan *et al*., 2011). It is not known whether this missense SNP is functional, but as this residue is conserved across most period homologues and is proximal to predicted Csnk1e target sites, it may directly underlie this association (Ebisawa *et al*., 2001).

We also found a nominally significant association between an *ARNTL2* SNP rs922270 and diurnal preference. This is the first time a SNP in the *ARNTL2* gene has been associated with diurnal preference. The C allele for this SNP had previously been associated with better afternoon/evening mood in a bipolar sample (Shi *et al*., 2008). As better afternoon and evening moods are associated with morning and evening preferences, respectively (Kerkhof, 1998), it is difficult to relate these findings to our own. *ARNTL2* was thought to be unnecessary for the core circadian clock, as its paralogue *Arntl* is the only circadian gene that when knocked out on its own causes arrhythmic locomotor activity in mice (Bunger *et al*., 2000). Recently, it has been found that *Arntl* knockout mice have decreased *Arntl2* mRNA expression and that the arrhythmic locomotor activity can be rescued by *Arntl2* if it is driven by a constitutively activated promoter (Shi *et al*., 2010). The ARNTL2 protein may play an important role in the molecular clock, and thus genetic variation in *ARNTL2* could directly impact diurnal preference by affecting the core molecular clock. As this SNP is intronic, and not known to affect splicing, it is possible that it is in linkage disequilibrium with another functional SNP that, in turn, may underlie this association.

The associations with *PER3* and *ARNTL2* were still significant in the combined analysis including all 10 SNPs, suggesting that they are at least partially independent. This analysis also found associations of SNPs in *FBXL3* and *GNβ3*, with diurnal preference not revealed in the individual analyses. For the *GNβ3* association the T allele was associated with increased morningness, which matches a previous study (Johansson *et al*., 2004), while this is the first association of a *FBXL3* SNP with diurnal preference in humans, although *Fbxl3* mutant mice are known to have an altered circadian period (Godinho *et al*., 2007).

### Global PSQI score

We found a novel significant association between the functional *GNβ3* SNP rs5443 and the global PSQI sleep quality score. This SNP is part of a haplotype that was associated previously with diurnal preference (Lee *et al*., 2007). Additionally, two other SNPs in this gene have been associated with increased wakefulness after sleep onset in an elderly population (Evans *et al*., 2013). Although this SNP does not cause an amino acid change, it has been shown to lead to alternative splicing, leading in turn to the shorter Gnβ3-s isoform which causes increased signal transduction (Siffert *et al*., 1998). This gene codes for the beta-subunit of the membrane-associated heterotrimeric guanine nucleotide binding proteins (G-proteins). G-proteins are integral to a number of secondary messenger pathways, including those for serotonin and noradrenaline receptors (Millan *et al*., 2008), both of which are involved in sleep mechanisms (Murillo-Rodriguez *et al*., 2012). It is thus possible that this SNP directly underlies these associations by altering the signalling pathways of one of these key neurotransporter systems.

### Sleep duration

Both the *PER2* SNP rs2304672 and the *GNβ3* SNP rs5443 were associated with sleep duration. Genetic variation in *PER2* has been associated previously with advanced sleep phase syndrome (ASPS) (Toh *et al*., 2001); additionally, *Per2* knockout mice have initially diminished delta activity in response to sleep deprivation (Kopp *et al*., 2002). Of the two large genome-wide association studies (GWAS) conducted for sleep measures, one found a nominally significant association of genetic variation in *PER2* with sleep duration (Allebrandt *et al*., 2013), while the other found that genetic variation in *PER3* was nominally associated with sleep duration and sleep latency (Byrne *et al*., 2013). Part of the discrepancy between these studies and our findings may be due to the fact that different questionnaires were used to measure subjective sleep characteristics. The *PER2* association is due to a rare genotype and thus may be due to chance, although it is worth noting that rare SNPs are thought to explain a portion of the heritability that is missing from GWAS studies (Dickson *et al*., 2010; Manolio *et al*., 2009).

Both these associations remained mainly unchanged in a combined analysis of these two SNPs, suggesting that two associations are independent of each other. This analysis also found an association between the *FBXL3* SNP with sleep duration not revealed in the individual analyses, which is the first reported association of *FBXL3* with sleep duration.

### Sleep latency

We found nominally significant associations of both *ARNTL2* and *DBP* with sleep latency. The effects of *ARNTL2* may be related to its potential role in regulating sleep timing discussed above. The *DBP* SNP was associated previously with worse evening mood (Shi *et al*., 2008). Interestingly, there is a wealth of evidence that *Dbp* gene expression decreases following sleep deprivation in both mice and rats (Curie *et al*., 2013; Franken *et al*., 2007; Wisor *et al*., 2002). These findings suggest that *DBP* may directly play a role in sleep homeostasis.

In the combined analysis, the *DBP* association remained, while the *ARNTL2* association disappeared. This suggests that the *ARNTL2* association is not independent of the *DBP* association.

### Sleep quality

Four SNPs were associated with sleep quality, two of which were very rare: *FBLX3* (*n* = 2) and *PER2* (*n* = 3). This is the first study suggesting that *FBXL3* may play a role in the regulation of sleep. Animal models with mutations in *Fbxl3* have a longer circadian period (Godinho *et al*., 2007), but no study has investigated this gene's role in sleep homeostasis. In the combined analysis only the *FBLX3* association was significant.

### Candidate gene association studies

Genome-wide association studies approaches demonstrate that there are likely to be a large number of genes that affect sleep measures, most of which have small effects. Candidate gene approaches can be useful in determining genetic variants with small effects as they do not require the same scale of multiple testing corrections required in GWAS approaches, but they have limitations (Lewis and Knight, 2012). Any candidate gene approach requires that the *a priori* rationale for its selection is valid. Additionally, as with GWAS studies, it is difficult to determine the causal variant underlying the association and significant associations may be false positives. Finally, any candidate gene association study needs to be replicated in additional and varied samples (Lewis and Knight, 2012).

In summary, we found that genetic variation in genes with circadian expression is associated with both diurnal preference (*PER3* and *ARNTL2)* and specific subjective sleep measures (*GNβ3*). These findings are in line with previous studies that found circadian gene involvement in sleep homeostasis (Franken, 2013; Franken and Dijk, 2009; Wisor *et al*., 2002), and stress the importance of further studies aimed at dissecting the specific roles that individual circadian genes play in these two interrelated but distinct behaviours. Further studies combining new technologies, such as next-generation sequencing that allows for detection of all SNPs, and the use of more refined phenotypic measurements, such as employing actigraphy or polysomnography, will be critical in dissecting the specific roles of circadian genes in these behaviours.
